# African Vaccination Week as a catalyst for integrated services and immunisation equity: synthesis of implementation insights from countries in the WHO African Region

**DOI:** 10.1136/bmjph-2025-004798

**Published:** 2026-05-16

**Authors:** Aschalew Teka Bekele, Crépin H Dadjo, Patricia E M Manga, Ado Mpia Bwaka, Yolande Vou Masembe, Sarah Wanyoike, Charles Shey Wiysonge

**Affiliations:** 1 Vaccine-Preventable Diseases Programme, World Health Organization Regional Office for Africa, Brazzaville, Congo; 2 Intercountry Support Team for West Africa, Vaccine-Preventable Diseases Programme, World Health Organization Regional Office for Africa, Ouagadougou, Burkina Faso; 3 Intercountry Support Team for Central Africa, Vaccine Preventable Diseases, World Health Organization Regional Office for Africa, Libreville, Brazzaville, Gabon; 4 Intercountry Support Team for East and Southern Africa, Vaccine Preventable Diseases, World Health Organization Regional Office for Africa, Harare, Zimbabwe; 5 South African Medical Research Council, Cochrane South Africa, Cape Town, South Africa; 6 Department of Global Health, Stellenbosch University, Cape Town, South Africa

**Keywords:** Vaccination, Public Health, Community Participation, Communication

## Abstract

**Introduction:**

The African Vaccination Week (AVW) is a coordinated initiative designed to raise awareness, strengthen immunisation delivery and promote equity across the WHO African Region. In 2025, AVW coincided with the Immunisation Agenda 2030 midpoint, offering a strategic opportunity to accelerate progress towards reaching zero-dose and under-vaccinated populations.

**Methods:**

We conducted a cross-sectional synthesis using aggregated secondary data from country implementation reports submitted to the WHO Regional Office for Africa and insights from postimplementation regional webinars. Data were analysed descriptively to summarise vaccination outputs, integrated service delivery and implementation innovations across countries.

**Results:**

25 countries reported implementing AVW 2025 activities. Approximately 11.5 million children were vaccinated, including 353 133 zero-dose children. Around 100 000 adults received vaccines, primarily tetanus–diphtheria and COVID-19 vaccines. Other interventions delivered along with vaccination included vitamin A supplementation and deworming for over 250 000 children. Several countries leveraged AVW 2025 to introduce vaccines, launch their national immunisation strategy and strengthen integration with primary healthcare services.

**Conclusions:**

The AVW 2025 functioned as a regional implementation platform for highlighting immunisation priorities, reaching missed children and supporting integrated primary healthcare delivery across the WHO African Region.

Country experiences indicate that AVW achieves the greatest impact when aligned with national programmes and embedded within routine planning processes. Variations in participation and reporting point to the need for stronger advance preparation, sustained engagement with all Member States and adaptable support tailored to country contexts. Positioning AVW as a feasible, low-opportunity-cost mechanism for advocacy, visibility and service delivery alignment can contribute meaningfully to broader immunisation and primary healthcare goals in the Region.

**WHAT IS ALREADY KNOWN ON THIS TOPIC**

- The African Vaccination Week (AVW), held during the last week of April, is a long-standing regional initiative that promotes immunisation awareness and delivery across the WHO African Region.

**WHAT THIS STUDY ADDS**

- This synthesis provides the first regional overview of AVW implementation at the midpoint of the Immunisation Agenda 2030, quantifying reach among zero-dose and undervaccinated children and documenting integration of immunisation services with broader primary healthcare services across 25 countries in the WHO African Region.

**HOW THIS STUDY MIGHT AFFECT RESEARCH, PRACTICE OR POLICY**

- The findings support institutionalising AVW within national immunisation calendars and budgets as a strong foundation for advocacy and stakeholder mobilisation towards advancing equitable, integrated service delivery.

## Introduction

 Immunisation remains one of the most impactful and cost-effective public health interventions globally, preventing an estimated 3.5 to 5 million deaths annually.^1^ However, despite these advances, the WHO African Region continues to face persistent challenges in reaching zero-dose and underimmunised children, particularly in fragile, underserved and remote communities.^2^ The African Vaccination Week (AVW), launched in 2011, serves as a regionally synchronised initiative to promote awareness, increase vaccine uptake and mobilise political and community support.^3 4^ Celebrated annually during the last week of April, AVW provides a strategic opportunity to deliver integrated health services while reinforcing country-led ownership of immunisation programmes.

Since its inception in 2011, AVW has contributed to the immunisation of over 180 million individuals across diverse age groups, including providing vitamin A to approximately 120 million and deworming tablets to about 100 million children.^3^,^6^ In addition, more than 42 million children have been screened for malnutrition during AVW.^3^,^6^ These achievements underscore the cumulative impact of AVW as a high-leverage platform that enables countries to coordinate interventions, build momentum for national campaigns, and sustain public interest in immunisation.

The 2025 edition of AVW (AVW 2025), held from 24 to 30 April 2025 under the theme ‘Immunisation for all is humanly possible’, coincided with the midpoint of the Immunisation Agenda 2030 (IA2030).^2 7^ This short report synthesises regional implementation data from AVW 2025 to assess its contribution to reaching children with integrated immunisation and primary healthcare (PHC) services in the WHO African Region.

WHO provided communication assets, guidance documents, themes and technical support to countries. WHO did not fund the implementation activities reported by countries; all activities were carried out using national resources or existing programme budgets. The only financial support provided by WHO was a modest fund contribution to launch the event in Guinea.

This report aims to describe activities implemented by Member States during the 2025 AVW and the contexts in which they occurred; it does not aim to evaluate performance or assess programme impact.

## Methods

We conducted a rapid cross-sectional regional programme synthesis using aggregated secondary data collected as part of routine AVW implementation and monitoring. Data sources included country reports submitted to the WHO Regional Office for Africa (AFRO), documentation from regional and subregional coordination teams, and insights from pre-AVW and post-AVW regional webinars convened to support planning, implementation and learning.

AVW 2025 was coordinated through a regional structure involving WHO AFRO, Member States and immunisation partners. All 47 Member States of the WHO African Region were invited to implement AVW activities. A regional coordination committee was established comprising regional office staff, inter-country support teams and country-level communication and immunisation focal persons. The regional committee held biweekly meetings to track progress across countries.

To capture country experiences, a semistructured planning and reporting template was disseminated, and reports were collected by the intercountry support teams and compiled at the regional level. Regular reminders were sent to the countries to share their progress. The regional working groups coordination platforms were used to inform and engage partners about the AVW activities and to ensure their support through existing country coordination mechanisms. A regional webinar was organised with countries before AVW for countries to share their progress updates, and a post-AVW webinar was conducted to share achievements using a semistructured presentation template.

Countries implemented activities through national Expanded Programme on Immunisation (EPI) coordination mechanisms, supported by microplanning and implementation at subnational levels. All reports were generated by the Ministry of Health and submitted to WHO country offices or regional offices. Quantitative analysis was done using Excel to compile number of beneficiaries reached by intervention type. Qualitative data were analysed to identify emerging themes and responses were categorised into thematic areas such as zero-dose, life course vaccination, campaign activities, with key highlights wextracted for each country. Additional analysis assessed the number of countries that integrated immunisation with other services with integration defined as co-planning, joint health education or co-administration of services.

## Results

The official regional launch of AVW 2025 in the WHO African Region took place in Guinea under the auspices of the country’s Minister of Health. At WHO AFRO, there was a week-long photo exhibition entitled ‘The History of Vaccination: A Journey of Hope’ as well as sporting activities in support of immunisation.

Of the 47 WHO Member States in the WHO African Region, 25 (53.2%) submitted reports on activities implemented during AVW 2025. In Chad, the Head of State issued a statement in support of AVW 2025; and in São Tomé and Príncipe, the First Lady delivered a message in support of AVW 2025. In most countries, AVW 2025 activities were officially launched by the national Minister of Health or a high-ranking representative of the minister.

Countries used the week to strengthen immunisation advocacy, identify and vaccinate zero-dose and under-vaccinated children and integrate additional health services (table 1). Communication campaigns conducted through mainstream and social media channels reached millions of community members across countries.

**Table 1:** Summary of AVW 2025 activities by thematic area for the 25 reporting countries

Country	Activity type	Thematic focus	Highlights
Ghana	Integrated campaign	Zero-dose, community engagement	AVW integrated with Child Health Promotion Week, including Vitamin A and deworming
Ethiopia	Integrated delivery	Zero-dose, Big Catch-Up	Launched integrated outreach combining measles, routine vaccines and Big Catch-Up
Tanzania	Campaign integration	TCV communication, community mobilisation	Pretested TCV communication tools, combined with AVW, with outreach
South Sudan	Multiple introductions	Remote communities, Big Catch-Up	Launched PCV, Rota and MR; reached remote areas via AVW
Cabo Verde	Adult vaccination	Life-course, influenza	Delivered influenza and hepatitis B vaccines for adults
Niger	Campaign launch	MR, integration	Integrated measles-rubella campaign with Big Catch-Up
Nigeria	Outreach	Big Catch-Up	Conducted outreach in priority LGAs as part of Big Catch-Up
Kenya	Awareness and testing	TCV, media	Pre-tested TCV materials, hosted panel discussions
South Africa	Routine + campaign	DTP boosters, Big Catch-Up	Expanded DTP booster coverage under AVW
Guinea	Outreach	Routine strengthening	Community mobilisation for routine immunisation
Malawi	Recognition	Polio end, partnerships	Recognised partners after ending polio outbreak
Gabon	Awareness	Zero-dose	Used AVW to sensitise caregivers and communities
Madagascar	Community campaign	Big Catch-Up	Launched Big Catch-Up in multiple districts
Mozambique	Outreach	PHC linkage	Linked AVW with primary health outreach
Eswatini	Mobilisation	AVW routine linkage	Engaged communities in under-served areas
Burundi	Communication	Routine coverage	Focused on awareness and visibility activities
Benin	Community campaign	Community awareness and Big Catch-Up	Implemented intensified routine immunisation capturing zero-dose and undervaccinated children
Sao Tome	Routine outreach	Zero-dose	Microplanning and outreach in remote districts
Angola	Integrated services	PHC + immunisation	Linked AVW with PHC revitalisation
Nigeria	Routine + campaign	Big catch-up	Big catch-up vaccination in targeted states
Chad	Launch + awareness	Multiantigen	Conducted multi-vaccine campaign with AVW visibility
DRC	Catch-up	Measles + routine	Integrated measles campaign with AVW mobilisation
The Gambia	Launch + awareness	Routine immunisation and life course vaccination	A launch ceremony with speakers from permanent secretary of health, WHO representative and deputy director of health services followed by community awareness activities.
Sierra Leone	Advocacy	PHC + immunisation	A high-level event hosted by vice president and Minister of Health along with senior officials from WHO, CDC, Gavi, UNICEF and others. Conducted outreach campaigns, catch-up vaccination, community engagement and addressing misinformation

AVW, African Vaccination Week; CDC, Center for Disease Control; LGAs, Local Government Agencies; MR, measles-rubella; PCV, pneumococcal conjugate vaccine; PHC, primary healthcare; TCV, typhoid conjugate vaccine.

Several countries leveraged AVW 2025 to advance broader health system objectives. Malawi launched a new National Immunisation Strategy and an EPI Field Manual during AVW 2025. In some countries, there was joint commemoration with World Malaria Day, and three countries launched malaria vaccine introductions or enhanced follow-up for children who missed subsequent malaria doses after starting vaccination. For example, Burundi used the week to support malaria vaccine rollout. Ghana integrated AVW 2025 with its Child Health Promotion Week, delivering immunisation alongside nutrition services, deworming and civil registration. In Sierra Leone, high-level AVW 2025 events engaged political leaders and partners to address misinformation and reinforce linkages between PHC and immunisation services.

Overall, approximately 11.5 million children received vaccinations during AVW 2025, including 353 133 zero-dose children and an estimated 8.6 million children vaccinated against measles and rubella. In addition, around 100 000 adults were vaccinated, including approximately 93 000 women who received tetanus–diphtheria vaccines and 6700 individuals who received COVID-19 vaccine doses. Integrated interventions delivered alongside immunisation included vitamin A supplementation and deworming for more than 250 000 children.

## Discussion

The 2025 edition of the AVW demonstrated the continued relevance of regionally coordinated implementation platforms in advancing immunisation goals. Alignment with IA2030 priorities—particularly reaching zero-dose children—enabled countries to use AVW 2025 as an accelerator for integrated service delivery, advocacy and policy engagement. The scale of reported vaccination outputs and the range of integrated interventions underscore AVW’s potential to strengthen collaboration among PHC stakeholders when embedded within national planning processes.

Institutionalisation emerged as a key enabler of sustainability. For example, Ghana integrated AVW 2025 into its Ministry of Health calendar and allocated dedicated resources, illustrating how AVW can transition from a partner-supported campaign to a nationally owned platform. AVW 2025 also catalysed new vaccine introductions and scale-up, including human papillomavirus and malaria vaccines in selected countries.

Many countries leveraged AVW to reinforce or expand activities already planned or underway, including campaigns, new vaccine introductions or community engagement efforts. This reflects a practical and feasible approach that aligns AVW with existing national priorities and minimises disruption to routine service delivery. However, it is critical to avoid creating additional stand-alone activities solely for commemorating the week when such activities require substantial new resources, pose feasibility challenges or increase opportunity costs. AVW is most useful when used to enhance visibility, coordination and advocacy rather than becoming a resource-intensive event.

Challenges remain, including variability in documentation quality and reliance on external funding may limit sustainability.^4^ Structural barriers related to geography, insecurity, and social exclusion also continue to constrain equitable access to immunisation services.^2^ Only 25 of 47 countries submitted implementation reports, introducing potential bias and limiting generalisability. For these reasons, the findings presented in this synthesis should be interpreted cautiously. Strengthened advance planning, systematic engagement and tailored support are needed to ensure AVW remains a feasible, low-opportunity-cost platform that reinforces ongoing immunisation and PHC activities.

## Limitations

This synthesis is based solely on country-reported implementation data submitted to WHO, which may vary in completeness, accuracy, and level of detail. Only 25 of 47 Member States provided reports, limiting regional representativeness and introducing potential reporting and interpretive bias. While WHO coordinated AVW, provided communication assets, guidance and limited media-related financial support, it was not involved in drafting country reports; however, institutional proximity to programme coordination may still influence interpretation. Differences in national documentation systems and the absence of external verification further constrain comparability across countries. Broader governance and incentive factors that shape reporting practices were not systematically assessed. For these reasons, the findings should be interpreted cautiously and viewed as a description of activities reported by participating countries rather than a comprehensive assessment of AVW implementation across the Region.

## Conclusions

The 2025 AVW, implemented at the midpoint of IA 2030, provided a shared regional platform that countries used to highlight immunisation priorities, reach missed children and deliver integrated primary services. Experiences by participating countries show that AVW can be most useful when aligned with existing national programmes and integrated within routine planning processes.

Variation in participation and reporting underscores the importance of strengthened advance planning, sustained engagement with all Member States, and adaptable support tailored to country contexts. Positioning AVW as a feasible, low-opportunity-cost mechanism for advocacy, visibility, and service delivery alignment may contribute to broader immunisation and PHC efforts across the Region.
